# Bayesian Cramér-Rao Lower Bounds for Prediction and Smoothing of Nonlinear TASD Systems

**DOI:** 10.3390/s22134667

**Published:** 2022-06-21

**Authors:** Xianqing Li, Zhansheng Duan, Qi Tang, Mahendra Mallick

**Affiliations:** 1 Center for Information Engineering Science Research, School of Automation Science and Engineering, Xi’an Jiaotong University, Xi’an 710049, China; lixianqing@stu.xjtu.edu.cn (X.L.); tangqi@stu.xjtu.edu.cn (Q.T.); 2 Independent Consultant, Anacortes, WA 98221, USA; mmallick.us@gmail.com

**Keywords:** Bayesian Cramér-Rao lower bound (BCRLB), two-adjacent-states dependent (TASD) measurements, autocorrelated noises, cross-correlated noises, prediction, smoothing

## Abstract

The performance evaluation of state estimators for nonlinear regular systems, in which the current measurement only depends on the current state directly, has been widely studied using the Bayesian Cramér-Rao lower bound (BCRLB). However, in practice, the measurements of many nonlinear systems are two-adjacent-states dependent (TASD) directly, i.e., the current measurement depends on the current state as well as the most recent previous state directly. In this paper, we first develop the recursive BCRLBs for the prediction and smoothing of nonlinear systems with TASD measurements. A comparison between the recursive BCRLBs for TASD systems and nonlinear regular systems is provided. Then, the recursive BCRLBs for the prediction and smoothing of two special types of TASD systems, in which the original measurement noises are autocorrelated or cross-correlated with the process noises at one time step apart, are presented, respectively. Illustrative examples in radar target tracking show the effectiveness of the proposed recursive BCRLBs for the prediction and smoothing of TASD systems.

## 1. Introduction

Filtering, prediction and smoothing have attracted wide attention in many engineering applications, such as target tracking [1,2], signal processing [3], sensor registration [4], econometrics forecasting [5], localization and navigation [6,7], etc. For filtering, the Kalman filter (KF) [8] is optimal for linear Gaussian systems in the sense of minimum mean squared error (MMSE). However, most real-world system models are usually nonlinear, which does not meet the assumptions of the Kalman filter. To deal with this, many nonlinear filters have been developed. The extended Kalman filter (EKF) [9] is the most well-known one, which approximates nonlinear systems as linear systems by the first-order Taylor series expansion of the nonlinear dynamic and/or measurement systems. The divided difference filter (DDF) was proposed in [10] using the Stirling interpolation formula. DDFs include the first-order divided difference filter (DD1) and second-order divided difference filter (DD2), depending on the interpolation order. Moreover, some other nonlinear filters have also been proposed, including the unscented Kalman filter (UKF) [11,12], quadrature Kalman filter (QKF) [13], cubature Kalman filter (CKF) [14,15], etc. All these nonlinear filters use different approximation techniques, such as function approximation and moment approximation [16]. Another type of nonlinear filter is the particle filter (PF) [17,18], which uses the sequential Monte Carlo method to generate random sample points to approximate the posterior density. Prediction is also very important since it can help people make decisions in advance and prevent unknown dangers. Following the same idea of filters, various predictors have been studied, e.g., Kalman predictor (KP) [19], extended Kalman predictor (EKP) [20], unscented Kalman predictor (UKP) [21], cubature Kalman predictor (CKP) [22] and particle predictor (PP) [23]. It is well known that smoothing is, in general, more accurate than the corresponding filtering. To achieve higher precision for estimation, many smoothers have been proposed, such as the Kalman smoother (KS) [24], extended Kalman smoother (EKS) [25], unscented Kalman smoother (UKS) [26], cubature Kalman smoother (CKS) [27] and particle smoother (PS) [28].

Despite the significant progress in nonlinear filtering, prediction and smoothing, they mainly deal with nonlinear regular dynamic systems, in which the current measurement depends only on the current state directly. However, in practice, many systems may have two-adjacent-states dependent (TASD) measurements. For example, the nonlinear systems having autocorrelated measurement noises or cross-correlated measurement and process noises at one time step apart [24] can be regarded as systems with TASD measurements. These types of systems are common in practice. For example, in many radar systems, the auto-correlations of measurement noises can not be ignored [29,30] due to the high measurement frequency. In satellite navigation systems, multi-path error and weak GPS signals make the measurement noise regarded as integral to white noise [31]. Further, in signal processing, measurement noises are usually autocorrelated because of time-varying fading and band-limited channel [32,33]. In sensor fusion, the time alignment of different sensors will cause the dependency of process noise and measurement noise [34]. In target-tracking systems, the discretization of continuous systems can induce the cross-correlation between the process and measurement noises at one time step apart [35]. In aircraft inertial navigation systems, the vibration of the aircraft has a common effect on the sources of the process and measurement noises, which results in the cross-correlation between them [36]. For these systems, some estimators have been studied. To deal with the nonlinear systems with autocorrelated measurement noise, which is modeled as a first-order autoregressive sequence, a nonlinear Gaussian filter and a nonlinear Gaussian smoother were proposed in [37,38], respectively. It makes the new measurement noise white by reformulating a TASD measurement equation. A PF was proposed for the nonlinear systems with dependent noise [39], in which the measurement is dependent on two adjacent states due to the cross-correlation between process and measurement noises. For nonlinear systems with the cross-correlated process and measurement noises at one time step apart, the Gaussian approximate filter and smoother were proposed in [40].

As is well known, assessing the performance of estimators is of great significance. The posterior Cramér-Rao lower bound (PCRLB) defined as the inverse of Fisher information matrix (FIM), also called Bayesian Cramér-Rao lower bound (BCRLB), provides a lower bound on the performance of estimators for nonlinear systems [41,42], Ch. 4 of [43]. In [44,45], a recursive BCRLB was developed for the filtering of nonlinear regular dynamic systems in which the current measurement is only dependent on the current state directly. Moreover, the BCRLBs for the prediction and smoothing of nonlinear regular dynamic systems was proposed in [45]. Compared with the conventional BCRLB, a new concept called conditional PCRLB (CPCRLB) was proposed in [46]. This CPCRLB is conditioned on the actual past measurements and provides an effective online performance bound for filters. In [47], another two CPCRLBs, i.e., A-CPCRLB and D-CPCRLB, were proposed. Since the auxiliary FIM is discarded, A-CPCRLB in [47] is more compact than the CPCRLB proposed in [46]. D-CPCRLB in [47] is not recursive and directly approximates the exact bound through numerical computations.

Some recent work has conducted a filtering performance assessment of TASD systems. In [48], a BCRLB was provided for the filtering of nonlinear systems with higher-order colored noises. Further, they presented the BCRLB for a special case in which the measurement model is driven by first-order autocorrelated Gaussian noises. In [49], the BCRLBs were proposed for the filtering of nonlinear systems with two types of dependence structures, of which the type II dependency can lead to TASD measurements. However, both of them did not generalize the BCRLB in [48,49] to the general form of TASD systems. In addition, the recursive BCRLBs for the prediction and smoothing of TASD systems were not covered in [48,49]. For the general form of TASD systems, a CPCRLB for filtering was developed in [50], which is dependent on the actual measurements. Compared with the BCRLB, this CPCRLB can provide performance evaluations for a particular nonlinear system’s state realization and better criteria for online sensor selection. In practice, the TASD systems sometimes may incorporate some unknown nonrandom parameters. For the performance evaluation of joint state and parameter estimation for nonlinear parametric TASD systems, a recursive joint CRLB (JCRLB) was studied in [51].

As equally important as CPCRLB is the BCRLB. It only depends on the structures and parameters of the dynamic model and measurement model but not the specific realization of measurement. As a result of this, BCRLBs can be computed offline. The BCRLB for the filtering of the general form of TASD systems has been obtained as a special case of the JCRLB in [51] when the parameter belongs to the empty set. However, the BCRLBs for the prediction and smoothing of the general form of TASD systems have not been studied yet. This paper aims to obtain the BCRLB for the prediction and smoothing of such nonlinear systems. First, we develop the recursive BCRLBs for the prediction and smoothing of general TASD systems. A comparison between the BCRLBs for TASD systems and regular systems is also made, and specific and simplified forms of the BCRLBs for additive Gaussian noise cases are provided. Second, we study specific BCRLBs for the prediction and smoothing of two special types of TASD systems, with autocorrelated measurement noises and cross-correlated process and measurement noises at one time step apart, respectively.

The rest of this paper is organized as follows. Section 2 formulates the BCRLB problem for nonlinear systems with TASD measurements. Section 3 develops the recursions of BCRLB for the prediction and smoothing of general TASD systems. Section 4 presents specific BCRLBs for two special types of nonlinear systems with TASD measurements. In Section 5, some illustrative examples in radar target tracking are provided to verify the effectiveness of the proposed BCRLBs. Section 6 concludes the paper.

## 2. Problem Formulation

Consider the following general discrete-time nonlinear systems with TASD measurements

(1) $$\begin{array}{ccc} x_{k+1} & = & f_{k}\left(x_{k},w_{k}\right) \end{array}$$

(2) $$\begin{array}{ccc} z_{k} & = & h_{k}\left(x_{k},x_{k-1},v_{k}\right) \end{array}$$

where $x_{k}\in R^{n}$ and $z_{k}\in R^{m}$ are the state and measurement at time *k*, respectively, the process noise $\langle w_{k}\rangle$ and the measurement noise $\langle v_{k}\rangle$ are mutually independent white sequences with probability density functions (PDFs) $p(w_{k})$ and $p(v_{k})$, respectively. We assume that the initial state $x_{0}$ is independent of the process and measurement noise sequences with PDF $p(x_{0})$.

**Definition** **1.** *Define $X^{k}={\left[x_{0}^{\prime },\cdots ,x_{k}^{\prime }\right]}^{\prime }$ and $Z^{k}={\left[z_{1}^{\prime },\cdots ,z_{k}^{\prime }\right]}^{\prime }$ as the accumulated state and measurement up to time k, respectively. The superscript “′” denotes the transpose of a vector or matrix.*

**Definition** **2.** *Define ${\hat{X}}^{j|k}$ and ${\hat{x}}_{j|k}$ as estimates of $X^{j}$ and $x_{j}$ given the measurement $Z^{k}$, respectively. ${\hat{x}}_{j|k}$ are state estimates for filtering, prediction and smoothing when j=k, j>k and j<k, respectively.*

**Definition** **3.** *The mean square error (MSE) of ${\hat{X}}^{j|k}$ is defined as*

$$M^{j|k}≜E\left[{\tilde{X}}^{j|k}{\left({\tilde{X}}^{j|k}\right)}^{\prime }\right]=\int_{R^{km}}\int_{R^{(j+1)n}}{\tilde{X}}^{j|k}{\left({\tilde{X}}^{j|k}\right)}^{\prime }p\left(X^{j},Z^{k}\right)dX^{j}dZ^{k}$$

*The MSE of ${\hat{x}}_{j|k}$ is defined as*

$$M_{j|k}≜E\left[{\tilde{x}}_{j|k}{\left({\tilde{x}}_{j|k}\right)}^{\prime }\right]=\int_{R^{km}}\int_{R^{n}}{\tilde{x}}_{j|k}{\left({\tilde{x}}_{j|k}\right)}^{\prime }p\left(x_{j},Z^{k}\right)dx_{j}dZ^{k}$$

*where ${\tilde{X}}^{j|k}=X^{j}-{\hat{X}}^{j|k}$ and ${\tilde{x}}_{j|k}=x_{j}-{\hat{x}}_{j|k}$ are the associated estimation errors, $p(X^{j},Z^{k})$ and $p(x_{j},Z^{k})$ are the joint PDFs. $M_{j|k}$ are MSEs for filtering, prediction and smoothing when j=k, j>k and j<k, respectively.*

**Definition** **4.** *Define the FIM $J^{j|k}$ about the accumulated state $X^{j}$ as*

$$\begin{array}{cc} J^{j|k} & ≜E[-\Delta_{X^{j}}^{X^{j}}\ln p\left(X^{j},Z^{k}\right)]\\ \; & =-\int_{R^{km}}\int_{R^{(j+1)n}}\left(\Delta_{X^{j}}^{X^{j}}\ln p\left(X^{j},Z^{k}\right)\right)p\left(X^{j},Z^{k}\right)dX^{j}dZ^{k} \end{array}$$

*where Δ denotes the second-order derivative operator, i.e., $\Delta_{a}^{b}=\nabla_{a}\nabla_{b}^{\prime }$, and ∇ denotes the gradient operator.*

**Lemma** **1.** *The MSE of ${\hat{X}}^{j|k}$ satisfying certain regularity conditions as in [41] is bounded from below by the inverse of $J^{j|k}$ as [41,45]*

$$M^{j|k}≜E\left[{\tilde{X}}^{j|k}{\left({\tilde{X}}^{j|k}\right)}^{\prime }\right]\geq {\left(J^{j|k}\right)}^{-1}$$

*where the inequality means that the difference $M^{j|k}-{\left(J^{j|k}\right)}^{-1}$ is a positive semidefinite matrix.*

**Definition** **5.** *Define $J_{j|k}^{-1}$ as the n×n right-lower block of ${\left(J^{j|k}\right)}^{-1}$ and $J_{j|k}$ as the FIM about $x_{j}$, where “n” is the dimension of the state $x_{k}$. $J_{j|k}$ are FIMs for filtering, prediction and smoothing when j=k, j>k and j<k, respectively.*

**Lemma** **2.** *The MSE of ${\hat{x}}_{j|k}$ satisfying certain regularity conditions as in [41] is bounded from below by the inverse of $J_{j|k}$ as [41,44]*

$$M_{j|k}≜E\left[{\tilde{x}}_{j|k}{\left({\tilde{x}}_{j|k}\right)}^{\prime }\right]\geq J_{j|k}^{-1}$$

Compared with regular systems, the measurement $z_{k}$ of the nonlinear systems (1) and (2) not only depends on the current state $x_{k}$ but also the most recent previous state $x_{k-1}$ directly. The main goal of this paper is to obtain the recursive FIMs $J_{j|k}$ for the prediction and smoothing of nonlinear TASD systems without manipulating the larger matrix $J^{j|k}$.

## 3. Recursive BCRLBs for Prediction and Smoothing

### 3.1. BCRLBs for General TASD Systems

For simplicity, the following notations are introduced in advance

(3) $$\left\{\begin{array}{c} D_{k+1}^{m,n}=E\left[-\Delta_{x_{n}}^{x_{m}}\ln p\left(x_{k+1}|x_{k}\right)\right]={\left(D_{k+1}^{n,m}\right)}^{\prime }\\ E_{k+1}^{m,n}=E\left[-\Delta_{x_{n}}^{x_{m}}\ln p\left(z_{k+1}|x_{k+1},x_{k}\right)\right]={\left(E_{k+1}^{n,m}\right)}^{\prime } \end{array}\right.$$

where m,n∈{k,k+1}, and $D_{0}^{0,0}=E\left[-\Delta_{x_{0}}^{x_{0}}\ln p\left(x_{0}\right)\right]$.

To initialize the recursion for FIMs of prediction and smoothing, the recursion of the FIM $J_{k|k}$ for filtering is required. This can be obtained from Corollary 3 of [51], as shown in the following lemma.

**Lemma** **3.** *The FIM $J_{k|k}$ for filtering obeys the following recursion [51]*

(4) $$\begin{array}{cc} J_{k+1|k+1} & =D_{k+1}^{k+1,k+1}+E_{k+1}^{k+1,k+1}-\left(D_{k+1}^{k,k+1}+E_{k+1}^{k,k+1}\right)\\ \; & \;\;\;\;\cdot {\left(D_{k+1}^{k,k}+E_{k+1}^{k,k}+J_{k|k}\right)}^{-1}\left(D_{k+1}^{k+1,k}+E_{k+1}^{k+1,k}\right) \end{array}$$

*with $J_{0|0}=D_{0}^{0,0}=E\left[-\Delta_{x_{0}}^{x_{0}}\ln p\left(x_{0}\right)\right]$.*

#### 3.1.1. BCRLB for Prediction

**Theorem** **1.** *The FIMs $J_{j+1|k}$ and $J_{j|k}$ are related to each other through*

(5) $$J_{j+1|k}=D_{j+1}^{j+1,j+1}-D_{j+1}^{j,j+1}{\left(D_{j+1}^{j,j}+J_{j|k}\right)}^{-1}D_{j+1}^{j+1,j}$$

*for j=k,k+1,k+2,⋯.*

**Proof.**  See Appendix A. □

Substituting j=k,k+1,⋯,k+m−1 into (5), the recursions of the FIM for *m*-step prediction can be obtained as

(6) $$\left\{\begin{array}{c} J_{k+1|k}=D_{k+1}^{k+1,k+1}-D_{k+1}^{k,k+1}{\left(D_{k+1}^{k,k}+J_{k|k}\right)}^{-1}D_{k+1}^{k+1,k}\\ J_{k+2|k}=D_{k+2}^{k+2,k+2}-D_{k+2}^{k+1,k+2}{\left(D_{k+2}^{k+1,k+1}+J_{k+1|k}\right)}^{-1}D_{k+2}^{k+2,k+1}\\ \;\;\;\;\;\;\;\;\;\;\;\;\;\;\;\;\;\;\;\;\;\;\;\;\;\;\vdots \\ J_{k+m|k}=D_{k+m}^{k+m,k+m}-D_{k+m}^{k+m-1,k+m}{\left(D_{k+m}^{k+m-1,k+m-1}+J_{k+m-1|k}\right)}^{-1}D_{k+m}^{k+m,k+m-1} \end{array}\right.$$

where m≥1.

#### 3.1.2. BCRLB for Smoothing

Let ${\hat{X}}^{k|k}={\left[{\hat{x}}_{0|k}^{\prime },\cdots ,{\hat{x}}_{j|k}^{\prime },{\hat{x}}_{k|k}^{\prime }\right]}^{\prime }$, 1⩽j⩽k−1 be an estimate of the accumulated state consisting of the smoothing estimates ${\hat{x}}_{0|k},{\hat{x}}_{1|k},\cdots ,{\hat{x}}_{k-1|k}$, and the filtering estimate ${\hat{x}}_{k|k}$. The MSE $M^{k|k}$ for ${\hat{X}}^{k|k}$ is bounded from below by the inverse of $J^{k|k}$. Thus ${\left(J^{k|k}\right)}^{-1}$ contains the smoothing BCRLBs $J_{j|k}^{-1}$, j=0,1,⋯,k−1, and filtering BCRLB $J_{k|k}^{-1}$ on its main diagonal. Then we have

(7) $$\begin{array}{cc} {\left(J^{k|k}\right)}^{-1} & =\left[\begin{array}{cccccc} J_{0|k}^{-1} & \; & & \; & & \\ \; & \ddots & \; & & \; & \\ \; & \; & J_{j|k}^{-1} & \; & & \\ \; & \; & & J_{j+1|k}^{-1} & \; & \\ \; & \; & & \; & \ddots & \\ \; & \; & & \; & & J_{k|k}^{-1} \end{array}\right]\\ \; & =\left[\begin{array}{cc} {\left[{\left(J^{k|k}\right)}^{-1}\right]}_{11} & \\ \; & {\left[{\left(J^{k|k}\right)}^{-1}\right]}_{22} \end{array}\right] \end{array}$$

where zero blocks have been left empty, ${\left[{\left(J^{k|k}\right)}^{-1}\right]}_{11}=\mathrm{diag}\left(J_{0|k}^{-1},\cdots ,J_{j|k}^{-1}\right)$, ${\left[{\left(J^{k|k}\right)}^{-1}\right]}_{22}=\mathrm{diag}\left(J_{j+1|k}^{-1},\cdots ,J_{k|k}^{-1}\right)$, and ‘diag’ denotes diagonal matrix [52].

**Theorem** **2.** *The FIM $J_{j|k}$ for smoothing can be recursively obtained as*

(8) $$\begin{array}{cc} J_{j|k} & =J_{j|j}+D_{j+1}^{j,j}+E_{j+1}^{j,j}-\left(D_{j+1}^{j+1,j}+E_{j+1}^{j+1,j}\right)(J_{j+1|k}\\ \; & \;\;\;\;+D_{j+1}^{j+1,j+1}+E_{j+1}^{j+1,j+1}-J_{j+1|j+1}{)}^{-1}\left(D_{j+1}^{j,j+1}+E_{j+1}^{j,j+1}\right) \end{array}$$

*for j=k−1,k−2,⋯,0. This backward recursion is initialized by the FIM $J_{k|k}$ for filtering.*

**Proof.**  See Appendix B. □

### 3.2. Comparison with the BCRLBs for Nonlinear Regular Systems

For nonlinear regular systems, measurement $z_{k}$ only depends on state $x_{k}$ directly, i.e., $z_{k}=h_{k}\left(x_{k},v_{k}\right)$. Clearly, nonlinear regular systems are special cases of nonlinear TASD systems (2) since

(9) $$\begin{array}{cc} z_{k} & =h_{k}\left(x_{k},v_{k}\right)\\ \; & =h_{k}\left(x_{k},v_{k}\right)+0\cdot x_{k-1}\\ \; & =h_{k}^{*}\left(x_{k},x_{k-1},v_{k}\right) \end{array}$$

As a result, the likelihood function $p(z_{j+1}|x_{j+1},x_{j})$ for TASD systems in (3) will be reduced to $p(z_{j+1}|x_{j+1})$ for regular systems. Correspondingly, $E_{j+1}^{j,j}$, $E_{j+1}^{j+1,j}$ and $E_{j+1}^{j+1,j+1}$ in (3) will be reduced to

(10) $$\left\{\begin{array}{cc} & E_{j+1}^{j,j}=E\left[-\Delta_{x_{j}}^{x_{j}}\ln p\left(z_{j+1}|x_{j+1},x_{j}\right)\right]=0\\ & E_{j+1}^{j+1,j}=E\left[-\Delta_{x_{j}}^{x_{j+1}}\ln p\left(z_{j+1}|x_{j+1},x_{j}\right)\right]=0\\ & E_{j+1}^{j+1,j+1}=E\left[-\Delta_{x_{j+1}}^{x_{j+1}}\ln p\left(z_{j+1}|x_{j+1},x_{j}\right)\right]\\ & \;\;\;\;\;\;\;\;\;\;\;\;\;\;=E[-\Delta_{x_{j+1}}^{x_{j+1}}\ln p\left(z_{j+1}|x_{j+1}\right)] \end{array}\right.$$

Substituting $E_{j+1}^{j,j}$, $E_{j+1}^{j+1,j}$ and $E_{j+1}^{j+1,j+1}$ in (10) into (8), the recursion of the FIM for smoothing of TASD systems will be reduced to

(11) $$\begin{array}{cc} J_{j|k} & =J_{j|j}+D_{j+1}^{j,j}-D_{j+1}^{j+1,j}{\left(J_{j+1|k}+D_{j+1}^{j+1,j+1}+E_{j+1}^{j+1,j+1}-J_{j+1|j+1}\right)}^{-1}D_{j+1}^{j,j+1} \end{array}$$

This is exactly the recursion of the FIM for smoothing of nonlinear regular systems in [45]. That is, the recursion of the FIM for the smoothing of nonlinear regular systems is a special case of the recursion of the FIM for the smoothing of nonlinear TASD systems.

For the FIM of prediction, it can be seen that the FIMs for prediction in (5) of TASD systems are governed by the same recursive equations as the FIMs for regular systems in [45], except that $J_{j|k}$, j=k,k+1,k+2,⋯, is different. This is because predictions for both TASD systems and regular systems only depend on the same dynamic Equation (1).

Next, we study specific and simplified BCRLBs for TASD systems with additive Gaussian noises.

### 3.3. BCRLBs for TASD Systems with Additive Gaussian Noise

Assume that the nonlinear systems (1) and (2) is driven by additive Gaussian noises as

(12) $$\begin{array}{ccc} x_{k+1} & = & f_{k}\left(x_{k}\right)+w_{k} \end{array}$$

(13) $$\begin{array}{ccc} z_{k} & = & h_{k}\left(x_{k},x_{k-1}\right)+v_{k} \end{array}$$

where $w_{k}∼N\left(0,Q_{k}\right)$, $v_{k}∼N\left(0,R_{k}\right)$ and the covariance matrices $Q_{k}$ and $R_{k}$ are invertible. Then the D’s and E’s of (3) used in the recursions of FIMs for prediction and smoothing will be simplified to

(14) $$\left\{\begin{array}{cc} & D_{k+1}^{k,k}=E\left\{\left[\nabla_{x_{k}}f_{k}^{\prime }\left(x_{k}\right)\right]Q_{k}^{-1}{\left[\nabla_{x_{k}}f_{k}^{\prime }\left(x_{k}\right)\right]}^{\prime }\right\}\\ & D_{k+1}^{k+1,k}=-E\left[\nabla_{x_{k}}f_{k}^{\prime }\left(x_{k}\right)\right]Q_{k}^{-1}\\ & D_{k+1}^{k+1,k+1}=Q_{k}^{-1}\\ & E_{k+1}^{k,k}=E\left\{\left[\nabla_{x_{k}}h_{k+1}^{\prime }\left(x_{k+1},x_{k}\right)\right]R_{k+1}^{-1}{\left[\nabla_{x_{k}}h_{k+1}^{\prime }\left(x_{k+1},x_{k}\right)\right]}^{\prime }\right\}\\ & E_{k+1}^{k+1,k}=E\left\{\left[\nabla_{x_{k}}h_{k+1}^{\prime }\left(x_{k+1},x_{k}\right)\right]R_{k+1}^{-1}{\left[\nabla_{x_{k+1}}h_{k+1}^{\prime }\left(x_{k+1},x_{k}\right)\right]}^{\prime }\right\}\\ & E_{k+1}^{k+1,k+1}=E\left\{\left[\nabla_{x_{k+1}}h_{k+1}^{\prime }\left(x_{k+1},x_{k}\right)\right]R_{k+1}^{-1}{\left[\nabla_{x_{k+1}}h_{k+1}^{\prime }\left(x_{k+1},x_{k}\right)\right]}^{\prime }\right\} \end{array}\right.$$

Assume that the systems (12) and (13) is further reduced to a linear Gaussian system as

(15) $$\begin{array}{ccc} x_{k+1} & = & F_{k}x_{k}+w_{k} \end{array}$$

(16) $$\begin{array}{ccc} z_{k} & = & H_{k}x_{k}+C_{k-1}x_{k-1}+v_{k} \end{array}$$

where $w_{k}∼N\left(0,Q_{k}\right)$, $v_{k}∼N\left(0,R_{k}\right)$ and the covariance matrices $Q_{k}$ and $R_{k}$ are invertible. Then the D’s and E’s of (3) used in the recursions of FIMs for prediction and smoothing will be further simplified to

(17) $$\left\{\begin{array}{cc} \; & D_{k+1}^{k,k}=F_{k}^{\prime }Q_{k}^{-1}F_{k}\\ \; & D_{k+1}^{k+1,k}=-F_{k}^{\prime }Q_{k}^{-1}\\ \; & D_{k+1}^{k+1,k+1}=Q_{k}^{-1}\\ \; & E_{k+1}^{k,k}=C_{k}^{\prime }R_{k+1}^{-1}C_{k}\\ \; & E_{k+1}^{k+1,k}=C_{k}^{\prime }R_{k+1}^{-1}H_{k+1}\\ \; & E_{k+1}^{k+1,k+1}=H_{k+1}^{\prime }R_{k+1}^{-1}H_{k+1} \end{array}\right.$$

**Remark 1.**  *If we rewrite the linear TASD systems (15) and (16) as the following augmented form*

(18) $$\left[\begin{array}{c} x_{k+1}\\ x_{k} \end{array}\right]=\left[\begin{array}{cc} F_{k} & \\ \; & F_{k-1} \end{array}\right]\left[\begin{array}{c} x_{k}\\ x_{k-1} \end{array}\right]+w_{k}^{*}$$

(19) $$z_{k}=\left[\begin{array}{cc} H_{k} & C_{k-1} \end{array}\right]\left[\begin{array}{c} x_{k}\\ x_{k-1} \end{array}\right]+v_{k}$$

* where zero blocks have been left empty and* $w_{k}^{*}={\left[w_{k}^{\prime },w_{k-1}^{\prime }\right]}^{\prime }$ *, then the process noise* $w_{k}^{*}$ *in (18) will be correlated with its adjacent noises* $w_{k-1}^{*}$ *and* $w_{k+1}^{*}$ *, but uncorrelated with* $\left\{w_{0}^{*},\cdots ,w_{k-2}^{*},w_{k+2}^{*},\cdots \right\}$ *. For this special type of linear system, how to obtain its BCRLBs is still unknown.*

## 4. Recursive BCRLBs for Two Special Types of Nonlinear TASD Systems

Two special types of nonlinear systems, in which the measurement noises are autocorrelated or cross-correlated with the process noises at one time step apart, can be deemed as nonlinear TASD systems described in (1) and (2). These two types of nonlinear systems are very common in many engineering applications. For example, in target-tracking systems, the high radar measurement frequency will result in autocorrelations of measurement noises [29] and the discretization of continuous systems can induce the cross-correlation between the process and measurement noises at one time step apart [35]. In navigation systems, the multi-path error and weak GPS signal will make measurement noises autocorrelated [31] and the effect caused by vibration on the aircraft may result in the cross-correlation between the process and measurement noises [36]. Next, specific recursive BCRLBs for the prediction and smoothing of these two systems are obtained by applying the above theorems in Section 3.

### 4.1. BCRLBs for Systems with Autocorrelated Measurement Noises

Consider the following nonlinear system

(20) $$\begin{array}{ccc} x_{k+1} & = & f_{k}\left(x_{k}\right)+w_{k} \end{array}$$

(21) $$\begin{array}{ccc} y_{k} & = & l_{k}\left(x_{k}\right)+e_{k} \end{array}$$

where $l_{k}$ is a nonlinear measurement function, $\langle e_{k}\rangle$ is autocorrelated measurement noise satisfying a first-order autoregressive (AR) model [38]

(22) $$e_{k}=\Psi_{k-1}e_{k-1}+\xi_{k-1}$$

where $\Psi_{k-1}$ is the known correlation parameter, the process noise $\langle w_{k}\rangle$ and the driven noise $\langle \xi_{k-1}\rangle$ are mutually independent white noise sequences, and both independent of the initial state $x_{0}$ as well.

To obtain the BCRLBs for the prediction and smoothing of nonlinear systems with autocorrelated measurement noises, a TASD measurement equation is first constructed by differencing two adjacent measurements as

(23) $$z_{k}=y_{k}-\Psi_{k-1}y_{k-1}$$

Then, we can get a pseudo measurement equation depending on two adjacent states as

(24) $$\begin{array}{ccc} z_{k} & = & l_{k}\left(x_{k}\right)-\Psi_{k-1}l_{k-1}\left(x_{k-1}\right)+e_{k}-\Psi_{k-1}e_{k-1}\\ & = & h_{k}\left(x_{k},x_{k-1}\right)+v_{k} \end{array}$$

where

$$\begin{array}{cc} h_{k}\left(x_{k},x_{k-1}\right) & =l_{k}\left(x_{k}\right)-\Psi_{k-1}l_{k-1}\left(x_{k-1}\right)\\ v_{k} & =\xi_{k-1} \end{array}$$

Clearly, the pseudo measurement noise $\langle v_{k}\rangle$ in (24) is white and independent of the process noise $\langle w_{k}\rangle$ and the initial state $x_{0}$.

From the above, we know that the systems (20)–(22) is equivalent to the TASD systems (20) and (24). Applying Theorems 1 and 2 to this TASD system, we can get the BCRLBs for the prediction and smoothing of nonlinear systems with autocorrelated measurement noises.

Next, we discuss some specific and simplified recursions of FIMs for the prediction and smoothing of nonlinear and linear systems with autocorrelated measurement noises when the noises are Gaussian.

**Theorem** **3.** *For the nonlinear systems (20)–(22), if the process noise $w_{k}∼N\left(0,Q_{k}\right)$ and the driven noise $\xi_{k}∼N\left(0,R_{k}\right)$, then the D’s and E’s of (3) used in the recursions of FIMs for prediction and smoothing will be simplified to*

(25) $$\left\{\begin{array}{cc} \; & D_{k+1}^{k,k}=E\left\{\left[\nabla_{x_{k}}f_{k}^{\prime }\left(x_{k}\right)\right]Q_{k}^{-1}{\left[\nabla_{x_{k}}f_{k}^{\prime }\left(x_{k}\right)\right]}^{\prime }\right\}\\ \; & D_{k+1}^{k+1,k}=-E\left[\nabla_{x_{k}}f_{k}^{\prime }\left(x_{k}\right)\right]Q_{k}^{-1}\\ \; & D_{k+1}^{k+1,k+1}=Q_{k}^{-1}\\ \; & E_{k+1}^{k,k}=E\left\{\left[\nabla_{x_{k}}l_{k}^{\prime }\left(x_{k}\right)\Psi_{k}^{\prime }\right]R_{k}^{-1}{\left[\nabla_{x_{k}}l_{k}^{\prime }\left(x_{k}\right)\Psi_{k}^{\prime }\right]}^{\prime }\right\}\\ \; & E_{k+1}^{k+1,k}=-E\left\{\left[\nabla_{x_{k}}l_{k}^{\prime }\left(x_{k}\right)\Psi_{k}^{\prime }\right]R_{k}^{-1}{\left[\nabla_{x_{k+1}}l_{k+1}^{\prime }\left(x_{k+1}\right)\right]}^{\prime }\right\}\\ \; & E_{k+1}^{k+1,k+1}=E\left\{\left[\nabla_{x_{k+1}}l_{k+1}^{\prime }\left(x_{k+1}\right)\right]R_{k}^{-1}{\left[\nabla_{x_{k+1}}l_{k+1}^{\prime }\left(x_{k+1}\right)\right]}^{\prime }\right\} \end{array}\right.$$

**Proof.**  See Appendix C. □

**Corollary** **1.** *Assume that the systems (20)–(22) is reduced to a linear Gaussian system as*

(26) $$\begin{array}{cc} x_{k+1} & =F_{k}x_{k}+w_{k} \end{array}$$

(27) $$\begin{array}{cc} y_{k} & =L_{k}x_{k}+e_{k} \end{array}$$

(28) $$\begin{array}{cc} e_{k} & =\Psi_{k-1}e_{k-1}+\xi_{k-1} \end{array}$$

*Then the D’s and E’s of (25) in Theorem 3 will be simplified to*

(29) $$\left\{\begin{array}{cc} \; & D_{k+1}^{k,k}=F_{k}^{\prime }Q_{k}^{-1}F_{k}\\ \; & D_{k+1}^{k+1,k}=-F_{k}^{\prime }Q_{k}^{-1}\\ \; & D_{k+1}^{k+1,k+1}=Q_{k}^{-1}\\ \; & E_{k+1}^{k,k}=L_{k}^{\prime }\Psi_{k}^{\prime }R_{k}^{-1}\Psi_{k}L_{k}\\ \; & E_{k+1}^{k+1,k}=-L_{k}^{\prime }\Psi_{k}^{\prime }R_{k}^{-1}L_{k+1}\\ \; & E_{k+1}^{k+1,k+1}=L_{k+1}^{\prime }R_{k}^{-1}L_{k+1} \end{array}\right.$$

**Theorem** **4.** *For the linear Gaussian systems (26)–(28) with autocorrelated measurement noises, the inverse of FIM $J_{k+m|k}$ for m-step prediction in Corollary 1 is equivalent to the MSE matrix $P_{k+m|k}$ of the optimal prediction, m≥1, i.e.,*

(30) $$\begin{array}{cc} P_{k+m|k} & =J_{k+m|k}^{-1} \end{array}$$

**Proof.**  See Appendix D. □

Since $P_{k+m|k}=J_{k+m|k}^{-1}$, m≥1, the optimal predictors can attain the BCRLBs for prediction proposed in Corollary 1, i.e., the optimal predictors are efficient estimators for the linear Gaussian systems (26)–(28) with autocorrelated measurement noises.

### 4.2. BCRLBs for Systems with Noises Cross-Correlated at One Time Step Apart

Consider the following nonlinear system

(31) $$\begin{array}{ccc} x_{k+1} & = & f_{k}\left(x_{k}\right)+w_{k} \end{array}$$

(32) $$\begin{array}{ccc} z_{k} & = & l_{k}\left(x_{k}\right)+e_{k} \end{array}$$

where $w_{k}∼N\left(0,Q_{k}\right)$, $e_{k}∼N\left(0,E_{k}\right)$ and they are cross-correlated at one time step apart [39], satisfying $E\left[w_{k}e_{j}^{\prime }\right]=U_{k}\delta_{k,j-1}$, where $\delta_{k,j-1}$ is the Kronecker delta function. Both $\langle w_{k}\rangle$ and $\langle e_{k}\rangle$ are independent of the initial state $x_{0}$.

To obtain the BCRLBs for the prediction and smoothing of nonlinear systems with noises cross-correlated at one time step apart, as in [50], a TASD measurement equation is constructed as

(33) $$\begin{array}{ccc} z_{k} & = & l_{k}\left(x_{k}\right)+e_{k}+G_{k}\left(x_{k}-f_{k-1}\left(x_{k-1}\right)-w_{k-1}\right)\\ & = & h_{k}\left(x_{k},x_{k-1}\right)+v_{k} \end{array}$$

where

$$\begin{array}{cc} h_{k}\left(x_{k},x_{k-1}\right) & =l_{k}\left(x_{k}\right)+G_{k}\left(x_{k}-f_{k-1}\left(x_{k-1}\right)\right)\\ v_{k} & =e_{k}-G_{k}w_{k-1}\\ G_{k} & =U_{k-1}^{\prime }Q_{k-1}^{-1} \end{array}$$

Clearly, the pseudo measurement noise $\langle v_{k}\rangle$ is uncorrelated with the process noise $\langle w_{k-1}\rangle$, and $E[v_{k}]=0$, $\mathrm{cov}\left(v_{k}\right)=R_{k}=E_{k}-U_{k-1}^{\prime }Q_{k-1}^{-1}U_{k-1}$.

**Proposition** **1.** *For the reconstructed TASD systems (31) and (33), $h_{k}\left(x_{k},x_{k-1}\right)$ is independent of the pseudo measurement noise $\langle v_{k}\rangle$.*

**Proof.**  First, from the assumption of noise independence, we know that $x_{k-1}$ is independent of $\langle e_{k}\rangle$ and $\langle w_{k-1}\rangle$. Therefore, it is obvious that $x_{k-1}$ is independent of $\langle v_{k}\rangle$. Second, because the state $x_{k}$ in $h_{k}\left(x_{k},x_{k-1}\right)$ is only determined by $\left\{x_{0},w_{0},\cdots ,w_{k-1}\right\}$, which is independent of $\langle v_{k}\rangle$, the state $x_{k}$ is independent of $\langle v_{k}\rangle$. Therefore, $h_{k}\left(x_{k},x_{k-1}\right)$ is independent of the pseudo measurement noise $\langle v_{k}\rangle$. This completes the proof. □

Proposition 1 shows that the reconstructed TASD systems (31) and (33) satisfies the independence assumption of the TASD systems in Section 2.

From the above, we know that the systems (31) and (32) is equivalent to the TASD systems (31) and (33). Applying Theorems 1 and reftheorem4 to this TASD system, the BCRLBs for the prediction and smoothing of nonlinear systems in which the measurement noise is cross-correlated with the process noise at one time step apart can be obtained.

Next, we discuss some specific and simplified recursions of FIMs for the prediction and smoothing of nonlinear and linear systems with Gaussian process and measurement noises cross-correlated at one time step apart.

**Theorem** **5.** *For the nonlinear systems (31)–(32), if the process noise $w_{k}∼N\left(0,Q_{k}\right)$ and the measurement noise $e_{k}∼N\left(0,E_{k}\right)$, then the D’s and E’s of (3) used in recursions of FIMs for prediction and smoothing will be simplified to*

(34) $$\left\{\begin{array}{cc} \; & D_{k+1}^{k,k}=E\left\{\left[\nabla_{x_{k}}f_{k}^{\prime }\left(x_{k}\right)\right]Q_{k}^{-1}{\left[\nabla_{x_{k}}f_{k}^{\prime }\left(x_{k}\right)\right]}^{\prime }\right\}\\ \; & D_{k+1}^{k+1,k}=-E\left[\nabla_{x_{k}}f_{k}^{\prime }\left(x_{k}\right)\right]Q_{k}^{-1}\\ \; & D_{k+1}^{k+1,k+1}=Q_{k}^{-1}\\ \; & E_{k+1}^{k,k}=E\left\{\left[\nabla_{x_{k}}f_{k}^{\prime }\left(x_{k}\right)G_{k+1}^{\prime }\right]R_{k+1}^{-1}{\left[\nabla_{x_{k}}f_{k}^{\prime }\left(x_{k}\right)G_{k+1}^{\prime }\right]}^{\prime }\right\}\\ \; & E_{k+1}^{k+1,k}=-E\left\{\left[\nabla_{x_{k}}f_{k}^{\prime }\left(x_{k}\right)G_{k+1}^{\prime }\right]R_{k+1}^{-1}{\left[\nabla_{x_{k+1}}l_{k+1}^{\prime }\left(x_{k+1}\right)+G_{k+1}^{\prime }\right]}^{\prime }\right\}\\ \; & E_{k+1}^{k+1,k+1}=E\left\{\left[\nabla_{x_{k+1}}l_{k+1}^{\prime }\left(x_{k+1}\right)+G_{k+1}^{\prime }\right]R_{k+1}^{-1}{\left[\nabla_{x_{k+1}}l_{k+1}^{\prime }\left(x_{k+1}\right)+G_{k+1}^{\prime }\right]}^{\prime }\right\} \end{array}\right.$$

**Corollary** **2.** *Assume that the systems (31)–(32) is reduced to a linear Gaussian system as*

(35) $$\begin{array}{ccc} x_{k+1} & = & F_{k}x_{k}+w_{k} \end{array}$$

(36) $$\begin{array}{ccc} z_{k} & = & L_{k}x_{k}+e_{k} \end{array}$$

*Then the D’s and E’s of (34) in Theorem 5 will be simplified to*

(37) $$\left\{\begin{array}{cc} \; & D_{k+1}^{k,k}=F_{k}^{\prime }Q_{k}^{-1}F_{k}\\ \; & D_{k+1}^{k+1,k}=-F_{k}^{\prime }Q_{k}^{-1}\\ \; & D_{k+1}^{k+1,k+1}=Q_{k}^{-1}\\ \; & E_{k+1}^{k,k}=F_{k}^{\prime }G_{k+1}^{\prime }R_{k+1}^{-1}G_{k+1}F_{k}\\ \; & E_{k+1}^{k+1,k}=-F_{k}^{\prime }G_{k+1}^{\prime }R_{k+1}^{-1}{\left(L_{k+1}^{\prime }+G_{k+1}^{\prime }\right)}^{\prime }\\ \; & E_{k+1}^{k+1,k+1}=\left(L_{k+1}^{\prime }+G_{k+1}^{\prime }\right)R_{k+1}^{-1}{\left(L_{k+1}^{\prime }+G_{k+1}^{\prime }\right)}^{\prime } \end{array}\right.$$

**Theorem** **6.** *For the linear Gaussian systems (35) and (36) with cross-correlated process and measurement noises at one time step apart, the inverse of FIM $J_{k+m|k}$ for m-step prediction in Corollary 2 is equivalent to the MSE matrix $P_{k+m|k}$ of the optimal prediction, m≥1, i.e.,*

(38) $$\begin{array}{cc} P_{k+m|k} & =J_{k+m|k}^{-1} \end{array}$$

**Proof.**  See Appendix E. □

Since $P_{k+m|k}=J_{k+m|k}^{-1}$, m≥1, the optimal predictors can attain the BCRLBs for prediction proposed in Corollary 2, i.e., the optimal predictors are efficient estimators for the linear Gaussian systems (35) and (36) with cross-correlated process and measurement noises at one time step apart.

## 5. Illustrative Examples

In this section, illustrative examples in radar target tracking are presented to demonstrate the effectiveness of the proposed recursive BCRLBs for the prediction and smoothing of nonlinear TASD systems.

Consider a target with nearly constant turn (NCT) motion in a 2D plane [14,40,48,53]. The target motion model is

(39) $$x_{k+1}=\left[\begin{array}{cccc} 1 & \frac{\sin\omega T}{\omega } & 0 & \frac{\cos\omega T-1}{\omega }\\ 0 & \cos\omega T & 0 & -\sin\omega T\\ 0 & \frac{1-\cos\omega T}{\omega } & 1 & \frac{\sin\omega T}{\omega }\\ 0 & \sin\omega T & 0 & \cos\omega T \end{array}\right]x_{k}+w_{k}$$

where $x_{k}={\left[x_{k},{\dot{x}}_{k},y_{k},{\dot{y}}_{k}\right]}^{\prime }$ is the state vector, T=1 s is the sampling interval, $\omega =2^{\circ }$s${}^{-1}$ is the turning rate and the process noise $w_{k}∼N\left(0,Q_{k}\right)$ with [53]

(40) $$\begin{array}{cc} \; & Q_{k}=S_{w}\left[\begin{array}{cccc} \frac{2(\omega T-\sin\omega T)}{\omega^{3}} & \frac{1-\cos\omega T}{\omega^{2}} & 0 & \frac{\left(\omega T-\sin\omega T\right)}{\omega^{2}}\\ \frac{1-\cos\omega T}{\omega^{2}} & T & -\frac{\left(\omega T-\sin\omega T\right)}{\omega^{2}} & 0\\ 0 & -\frac{\left(\omega T-\sin\omega T\right)}{\omega^{2}} & \frac{2(\omega T-\sin\omega T)}{\omega^{3}} & \frac{1-\cos\omega T}{\omega^{2}}\\ \frac{\left(\omega T-\sin\omega T\right)}{\omega^{2}} & 0 & \frac{1-\cos\omega T}{\omega^{2}} & T \end{array}\right] \end{array}$$

where $S_{w}=0.1$ m${}^{2}$s${}^{-3}$ is the power spectral density.

Assume that a 2D radar is located at the origin of the plane. The measurement model is

(41) $$z_{k+1}=\left[\begin{array}{c} r_{k+1}^{m}\\ \theta_{k+1}^{m} \end{array}\right]=\left[\begin{array}{c} \sqrt{x_{k+1}^{2}+y_{k+1}^{2}}\\ \tan^{-1}\left(y_{k+1},x_{k+1}\right) \end{array}\right]+e_{k+1}$$

where the radar measurement vector $z_{k+1}$ is composed of the range measurement $r_{k+1}^{m}$ and bearing measurement $\theta_{k+1}^{m}$, and $e_{k+1}$ is the measurement noise.

### 5.1. Example 1: Autocorrelated Measurement Noises

In this example, we assume that the measurement noise sequence $\langle e_{k+1}\rangle$ in (41) is first-order autocorrelated and modeled as

(42) $$e_{k+1}=0.4Ie_{k}+\xi_{k}$$

where I is a 2×2 identity matrix, the driven noise $\xi_{k}∼N\left(0,R_{k}\right)$ with $R_{k}=$ diag$\left(\sigma_{r}^{2}\left(\xi \right),\sigma_{\theta }^{2}\left(\xi \right)\right)$, $\sigma_{r}\left(\xi \right)=30$ m and $\sigma_{\theta }\left(\xi \right)=30$ mrad. Further, $\langle w_{k}\rangle$ and $\langle \xi_{k}\rangle$ are mutually independent. The initial state $X_{0}∼N\left({\bar{X}}_{0},P_{0}\right)$ with

$$\begin{array}{cc} {\bar{X}}_{0} & ={\left[1000\;m,120\;{\mathrm{ms}}^{-1},1000\;m,0\;{\mathrm{ms}}^{-1}\right]}^{\prime }\\ P_{0} & =\mathrm{diag}(10,000\;m^{2},100\;m^{2}s^{-2},10,000\;m^{2},10\;m^{2}s^{-2}) \end{array}$$

To show the effectiveness of the proposed BCRLBs in this radar target tracking example with autocorrelated measurement noises, we use the cubature Kalman filter (CKF) [37], cubature Kalman predictor (CKP) [37] and cubature Kalman smoother (CKS) [38] to obtain the state estimates. These estimators generate an augmented measurement to decorrelate the autocorrelated measurement noises instead of using the first-order linearization method. Meanwhile, these Gaussian approximate estimators can obtain accurate estimates with very low computational cost, especially in the high-dimensional case with additive Gaussian noises. The RMSEs and BCRLBs are obtained over 500 Monte Carlo runs.

**Figure 1:**
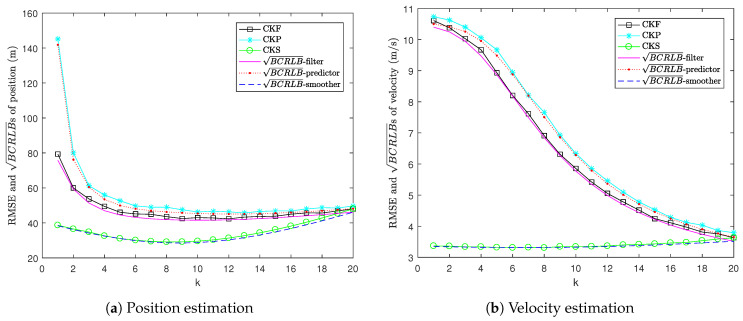
RMSE versus $\sqrt{\mathrm{BCRLB}}$ in Example 1.

Figure 1 shows the RMSE versus $\sqrt{\mathrm{BCRLB}}$ for position and velocity estimation. It can be seen that the proposed BCRLBs provide lower bounds to the MSEs of CKP and CKS. Moreover, the gaps between the RMSEs of CKP and CKS and the $\sqrt{\mathrm{BCRLB}}$s for one-step prediction and fixed-interval smoothing are very small. This means that the CKP and CKS are close to being efficient. Moreover, it can be seen that the $\sqrt{\mathrm{BCRLB}}$ for one-step prediction lies above the $\sqrt{\mathrm{BCRLB}}$ for filtering and the RMSE of CKP lies above the RMSE of CKF. This is because prediction only depends on the dynamic model, whereas filtering depends on both the dynamic and measurement models. Since smoothing uses both past and future information, the $\sqrt{\mathrm{BCRLB}}$ for fixed-interval smoothing is lower than the $\sqrt{\mathrm{BCRLB}}$ for filtering and the RMSE of CKS is lower than the RMSE of CKF.

**Figure 2:**
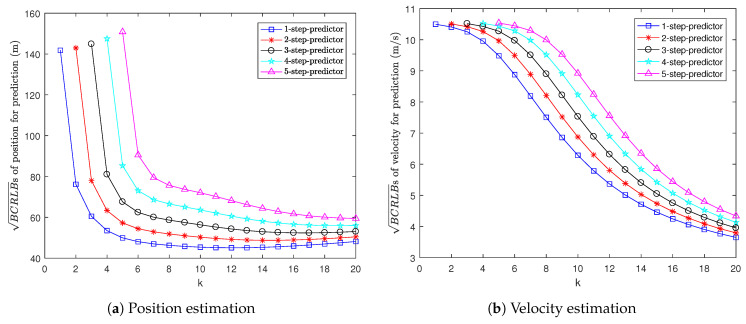
$\sqrt{\mathrm{BCRLB}}$s for prediction in Example 1.

Figure 2 shows the $\sqrt{\mathrm{BCRLB}}$s for multi-step prediction, i.e., 1-step to 5-step prediction. It can be seen that the more steps we predict ahead, the larger the $\sqrt{\mathrm{BCRLB}}$ for prediction is. This is because if we take more prediction steps, the predictions for position and velocity will be less accurate.

**Figure 3:**
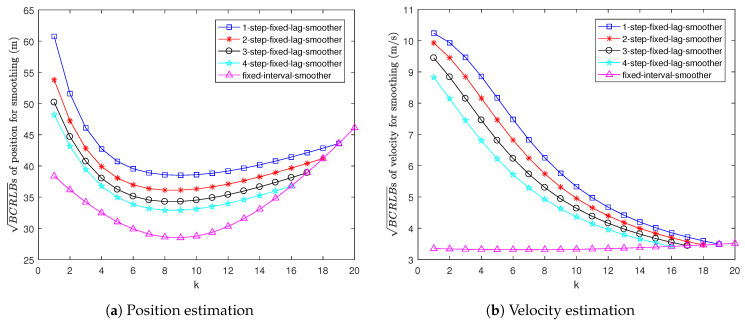
$\sqrt{\mathrm{BCRLB}}$s for smoothing in Example 1.

Figure 3 shows the $\sqrt{\mathrm{BCRLB}}$s for fixed-lag and fixed-interval smoothing. It can be seen that the $\sqrt{\mathrm{BCRLB}}$ for 1-step fixed-lag smoothing is the worst and the $\sqrt{\mathrm{BCRLB}}$ for fixed-interval smoothing is the best. This is because the smoothing estimation becomes more and more accurate as the length of the data interval increases.

### 5.2. Example 2: Cross-Correlated Process and Measurement Noises at One Time Step Apart

In this example, we assume that the process noise sequence $\langle w_{k}\rangle$ in (39) is cross-correlated with the measurement noise sequence $\langle e_{k}\rangle$ in (41) at one time step apart. The cross-correlation covariance is $E\left[w_{k}e_{k+1}^{\prime }\right]=U_{k}={\left[\begin{array}{cccc} 0.5 & 0.5 & 0.3 & 0.3\\ 0 & 0 & 0 & 0 \end{array}\right]}^{\prime }$. The distribution of $e_{k}$ is $N\left(0,E_{k}\right)$ with $E_{k}=$ diag$\left(\sigma_{r}^{2}\left(e\right),\sigma_{\theta }^{2}\left(e\right)\right)$, $\sigma_{r}\left(e\right)=30$ m and $\sigma_{\theta }\left(e\right)=40$ mrad. The initial state $X_{0}∼N\left({\bar{X}}_{0},P_{0}\right)$ with

$$\begin{array}{cc} {\bar{X}}_{0} & ={\left[1000\;m,120\;{\mathrm{ms}}^{-1},1000\;m,10\;{\mathrm{ms}}^{-1}\right]}^{\prime }\\ P_{0} & =\mathrm{diag}(10,000\;m^{2},1000\;m^{2}s^{-2},10,000\;m^{2},10\;m^{2}s^{-2}) \end{array}$$

To show the effectiveness of the proposed BCRLBs in this radar target tracking example with the the cross-correlated process and measurement noises at one time step apart, we use the cubature Kalman filter (CKF), cubature Kalman predictor (CKP) and cubature Kalman smoother (CKS) in [40] to obtain the state estimates. These estimators decorrelate the cross-correlation between process and measurement noises by reconstructing a pseudo measurement equation. Compared with the Monte Carlo approximation method, these Gaussian approximate estimators can give an effective balance between estimation accuracy and computational cost. A total of 500 Monte Carlo runs are performed to obtain the RMSEs and BCRLBs.

**Figure 4:**
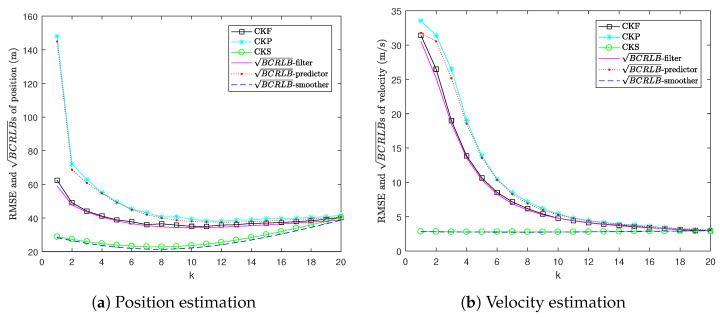
RMSE versus $\sqrt{\mathrm{BCRLB}}$ in Example 2.

Figure 4 shows the RMSEs of CKF, CKP and CKS versus three types of $\sqrt{\mathrm{BCRLB}}$s, i.e., for filtering, one-step prediction and fixed-interval smoothing. It can be seen that the RMSEs of CKP and CKS are bounded from below by their corresponding $\sqrt{\mathrm{BCRLB}}$s. It can also be observed that the gaps between the RMSEs of CKP and CKS and their corresponding $\sqrt{\mathrm{BCRLB}}$s are very small. This indicates that these estimators are close to being efficient. Moreover, we can see that the $\sqrt{\mathrm{BCRLB}}$ for one-step prediction lies above the $\sqrt{\mathrm{BCRLB}}$ for filtering, and the RMSE of CKP lies above the RMSE of CKF because prediction uses less information than filtering. Since smoothing uses data within the whole interval, the $\sqrt{\mathrm{BCRLB}}$ for fixed-interval smoothing is lower than the $\sqrt{\mathrm{BCRLB}}$ for filtering and the RMSE of CKS is lower than the RMSE of CKF.

**Figure 5:**
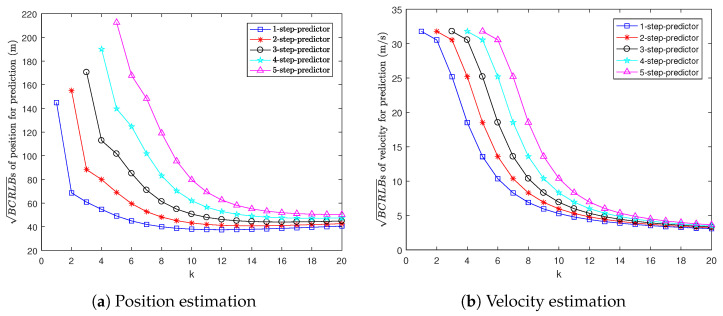
$\sqrt{\mathrm{BCRLB}}$s for prediction in Example 2.

Figure 5 shows the $\sqrt{\mathrm{BCRLB}}$s for multi-step prediction. We can see that the $\sqrt{\mathrm{BCRLB}}$ for prediction grows as the prediction step increases. This is because if we predict more steps ahead, the predictions for position and velocity will be less accurate.

**Figure 6:**
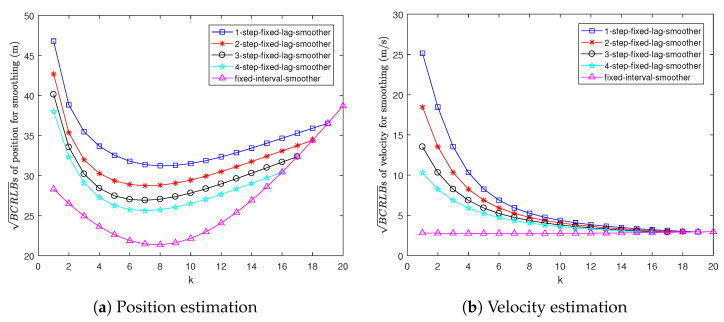
$\sqrt{\mathrm{BCRLB}}$s for smoothing in Example 2.

Figure 6 shows the $\sqrt{\mathrm{BCRLB}}$s for fixed-lag and fixed-interval smoothing. Clearly, smoothing becomes more accurate as the length of the data interval increases. Hence, the $\sqrt{\mathrm{BCRLB}}$ for 1-step fixed-lag smoothing is the worst. In contrast, the $\sqrt{\mathrm{BCRLB}}$ for fixed-interval smoothing is the best.

## 6. Conclusions

In this paper, we have proposed recursive BCRLBs for the prediction and smoothing of nonlinear dynamic systems with TASD measurements, i.e., the current measurement depends on both the current and the most recent previous state directly. A comparison with the recursive BCRLBs for nonlinear regular systems, in which the current measurement only depends on the current state directly, has been made. It is found that the BCRLB for the smoothing of regular systems is a special case of the newly proposed BCRLB, and the recursive BCRLBs for the prediction of TASD systems have the same forms as the BCRLBs for the prediction of regular systems except that the FIMs are different. This is because prediction only depends on the dynamic model, which is the same for both of them. Specific and simplified forms of the BCRLBs for the additive Gaussian noise cases have also been given. In addition, the recursive BCRLBs for the prediction and smoothing of two special types of nonlinear systems with TASD measurements, in which the original measurement noises are autocorrelated or cross-correlated with the process noises at one time step apart, have been presented, respectively. It is proven that the optimal linear predictors are efficient estimators if these two special types of nonlinear TASD systems are linear Gaussian.

## Data Availability

The authors declare that the data that support the findings of this study are available from the authors upon request.

## Abbreviations

The following abbreviations are used in this manuscript:

BCRLB	Bayesian Cramér-Rao lower bound
CPCRLB	conditional posterior Cramér-Rao lower bound
CKF	cubature Kalman filter
CKP	cubature Kalman predictor
CKS	cubature Kalman smoother
FIM	Fisher information matrix
MSE	mean square error
MMSE	minimum mean squared error
JCRLB	joint Cramér-Rao lower bound
PCRLB	posterior Cramér-Rao lower bound
PDF	probability density function
RMSE	root mean square error
TASD	Two-adjacent-states dependent

## Appendix A. Proof of Theorem 1

For the FIM $J^{j+1|k}$, the joint PDF of $X^{j+1}$ and $Z^{k}$ is

(A1) $$\begin{array}{cc} p_{k}^{j+1}≜p\left(X^{j+1},Z^{k}\right) & =p\left(X^{j},Z^{k}\right)p\left(x_{j+1}|X^{j},Z^{k}\right)\\ \; & =p_{k}^{j}p\left(x_{j+1}|x_{j}\right) \end{array}$$

Partition $X^{j}$ as $X^{j}={\left[{\left(X^{j-1}\right)}^{\prime },x_{j}^{\prime }\right]}^{\prime }$ and $J^{j|k}$ as

(A2) $$\begin{array}{ccc} J^{j|k} & = & -E_{p_{k}^{j}}\left[\begin{array}{cc} \Delta_{X^{j-1}}^{X^{j-1}}\ln p_{k}^{j} & \Delta_{X^{j-1}}^{x_{j}}\ln p_{k}^{j}\\ \Delta_{x_{j}}^{X^{j-1}}\ln p_{k}^{j} & \Delta_{x_{j}}^{x_{j}}\ln p_{k}^{j} \end{array}\right]\\ & = & \left[\begin{array}{cc} J_{j|k}^{11} & J_{j|k}^{12}\\ J_{j|k}^{21} & J_{j|k}^{22} \end{array}\right] \end{array}$$

Since $J_{j|k}^{-1}$ is equal to the n×n right-lower block of ${\left(J^{j|k}\right)}^{-1}$, from the inversion of a partitioned matrix [24], the FIM about $x_{j}$ can be obtained as

(A3) $$J_{j|k}=J_{j|k}^{22}-J_{j|k}^{21}{\left(J_{j|k}^{11}\right)}^{-1}J_{j|k}^{12}$$

Partition $X^{j+1}$ as $X^{j+1}={\left[{\left(X^{j-1}\right)}^{\prime },x_{j}^{\prime },x_{j+1}^{\prime }\right]}^{\prime }$ and $J^{j+1|k}$ as

(A4) $$\begin{array}{cc} J^{j+1|k} & =-E_{p_{k}^{j+1}}\left[\begin{array}{ccc} \Delta_{X^{j-1}}^{X^{j-1}}\ln p_{k}^{j+1} & \Delta_{X^{j-1}}^{x_{j}}\ln p_{k}^{j+1} & \Delta_{X^{j-1}}^{x_{j+1}}\ln p_{k}^{j+1}\\ \Delta_{x_{j}}^{X^{j-1}}\ln p_{k}^{j+1} & \Delta_{x_{j}}^{x_{j}}\ln p_{k}^{j+1} & \Delta_{x_{j}}^{x_{j+1}}\ln p_{k}^{j+1}\\ \Delta_{x_{j+1}}^{X^{j-1}}\ln p_{k}^{j+1} & \Delta_{x_{j+1}}^{x_{j}}\ln p_{k}^{j+1} & \Delta_{x_{j+1}}^{x_{j+1}}\ln p_{k}^{j+1} \end{array}\right] \end{array}$$

where

(A5) $$\begin{array}{cc} E_{p_{k}^{j+1}}\left(-\Delta_{X^{j-1}}^{X^{j-1}}\ln p_{k}^{j+1}\right) & =-\int_{R^{km}}\int_{R^{(j+2)n}}p_{k}^{j+1}\left(\Delta_{X^{j-1}}^{X^{j-1}}\ln p_{k}^{j+1}\right)dX^{j+1}dZ^{k}\\ \; & =-\int_{R^{km}}\int_{R^{(j+2)n}}p_{k}^{j}p\left(x_{j+1}|x_{j}\right)\left[\Delta_{X^{j-1}}^{X^{j-1}}\left(\ln p_{k}^{j}+\ln p\left(x_{j+1}|x_{j}\right)\right)\right]dX^{j+1}dZ^{k}\\ \; & =-\int_{R^{km}}\int_{R^{(j+1)n}}p_{k}^{j}\left(\Delta_{X^{j-1}}^{X^{j-1}}\ln p_{k}^{j}\right)dX^{j}dZ^{k}\\ \; & \overset{\left(A2\right)}{=}J_{j|k}^{11} \end{array}$$

Similarly, we can obtain

(A6) $$\left\{\begin{array}{cc} \; & E_{p_{k}^{j+1}}\left(-\Delta_{X^{j-1}}^{x_{j}}\ln p_{k}^{j+1}\right)=J_{j|k}^{12}\\ \; & E_{p_{k}^{j+1}}\left(-\Delta_{x_{j}}^{x_{j}}\ln p_{k}^{j+1}\right)=J_{j|k}^{22}+D_{j+1}^{j,j}\\ \; & E_{p_{k}^{j+1}}\left(-\Delta_{X^{j-1}}^{x_{j+1}}\ln p_{k}^{j+1}\right)=0\\ \; & E_{p_{k}^{j+1}}\left(-\Delta_{x_{j}}^{x_{j+1}}\ln p_{k}^{j+1}\right)=D_{j+1}^{j+1,j}\\ \; & E_{p_{k}^{j+1}}\left(-\Delta_{x_{j+1}}^{x_{j+1}}\ln p_{k}^{j+1}\right)=D_{j+1}^{j+1,j+1} \end{array}\right.$$

Then, $J^{j+1|k}$ can be rewritten as

(A7) $$J^{j+1|k}=\left[\begin{array}{ccc} J_{j|k}^{11} & J_{j|k}^{12} & 0\\ J_{j|k}^{21} & J_{j|k}^{22}+D_{j+1}^{j,j} & D_{j+1}^{j+1,j}\\ 0 & D_{j+1}^{j,j+1} & D_{j+1}^{j+1,j+1} \end{array}\right]$$

Since the prediction FIM matrix $J_{j+1|k}$ is the inverse of the right-lower n×n submatrix of $J^{j+1|k}$, from (A7), we have

$$\begin{array}{cc} J_{j+1|k} & =D_{j+1}^{j+1,j+1}-\left[\begin{array}{cc} 0 & D_{j+1}^{j,j+1} \end{array}\right]{\left[\begin{array}{cc} J_{j|k}^{11} & J_{j|k}^{12}\\ J_{j|k}^{21} & J_{j|k}^{22}+D_{j+1}^{j,j} \end{array}\right]}^{-1}\left[\begin{array}{c} 0\\ D_{j+1}^{j+1,j} \end{array}\right]\\ \; & =D_{j+1}^{j+1,j+1}-D_{j+1}^{j,j+1}{\left[J_{j|k}^{22}+D_{j+1}^{j,j}-J_{j|k}^{21}{\left(J_{j|k}^{11}\right)}^{-1}J_{j|k}^{12}\right]}^{-1}D_{j+1}^{j+1,j}\\ \; & \overset{\left(A3\right)}{=}D_{j+1}^{j+1,j+1}-D_{j+1}^{j,j+1}{\left(D_{j+1}^{j,j}+J_{j|k}\right)}^{-1}D_{j+1}^{j+1,j} \end{array}$$

This completes the proof.

## Appendix B. Proof of Theorem 2

For the FIM $J^{k|k}$, the joint PDF of $X^{k}$ and $Z^{k}$ at arbitrary time *k* is

(A8) $$\begin{array}{cc} p(X^{k},Z^{k}) & =p\left(X^{k-1},Z^{k-1}\right)p\left(x_{k}|X^{k-1},Z^{k-1}\right)p\left(z_{k}|x_{k},X^{k-1},Z^{k-1}\right)\\ \; & =p\left(X^{k-1},Z^{k-1}\right)p\left(x_{k}|x_{k-1}\right)p\left(z_{k}|x_{k},x_{k-1}\right) \end{array}$$

Similar to (A7), by using (A8), we can partition $J^{k|k}$ as

(A9) $$\begin{array}{cc} J^{k|k} & =\left[\begin{array}{cc} T_{j|j} & S_{j,k}\\ S_{j,k}^{\prime } & \Psi_{j,k} \end{array}\right]\\ \; & =\left[\begin{array}{ccccccc} K_{0} & N_{1} & \; & & \; & & \\ N_{1}^{\prime } & \ddots & \ddots & \; & & \; & \\ \; & \ddots & K_{j} & N_{j+1} & \; & & \\ \; & \; & N_{j+1}^{\prime } & K_{j+1} & \ddots & \; & \\ \; & \; & & \ddots & \ddots & \ddots & \\ \; & \; & & \; & \ddots & K_{k-1} & N_{k}\\ \; & \; & & \; & & N_{k}^{\prime } & D_{k}^{k,k}+E_{k}^{k,k} \end{array}\right] \end{array}$$

where zero blocks have been left empty, $K_{k}=D_{k}^{k,k}+E_{k}^{k,k}+D_{k+1}^{k,k}+E_{k+1}^{k,k}$, $N_{k}=D_{k}^{k,k-1}+E_{k}^{k,k-1}$, and the block matrix $T_{j|j}$ is

(A10) $$T_{j|j}=\left[\begin{array}{cc} J_{j|j}^{11} & J_{j|j}^{12}\\ J_{j|j}^{21} & J_{j|j}^{22}+D_{j+1}^{j,j}+E_{j+1}^{j,j} \end{array}\right]$$

Since $J_{j|k}^{-1}$ is the lower-right block of ${\left[{\left(J^{k|k}\right)}^{-1}\right]}_{11}$ defined in (7), we have

(A11) $$J_{j|k}^{-1}=\left[0,I_{n}\right]{\left[{\left(J^{k|k}\right)}^{-1}\right]}_{11}{\left[0,I_{n}\right]}^{\prime }$$

From (A9) and the inversion of a partitioned matrix [24], we have

(A12) $${\left[{\left(J^{k|k}\right)}^{-1}\right]}_{11}=T_{j|j}^{-1}+T_{j|j}^{-1}S_{j,k}{\left[{\left(J^{k|k}\right)}^{-1}\right]}_{22}S_{j,k}^{\prime }T_{j|j}^{-1}$$

and

(A13) $$\begin{array}{cc} \left[0,I_{n}\right]T_{j|j}^{-1}{\left[0,I_{n}\right]}^{\prime }\; & ={\left(J_{j|j}^{22}+D_{j+1}^{j,j}+E_{j+1}^{j,j}-J_{j|j}^{21}{\left(J_{j|j}^{11}\right)}^{-1}J_{j|j}^{12}\right)}^{-1}\\ \; & ={\left(J_{j|j}+D_{j+1}^{j,j}+E_{j+1}^{j,j}\right)}^{-1} \end{array}$$

Substituting (A12) into (A11) and using (A13) yields

(A14) $$\begin{array}{cc} J_{j|k}^{-1} & ={\left(J_{j|j}+D_{j+1}^{j,j}+E_{j+1}^{j,j}\right)}^{-1}+{\left(J_{j|j}+D_{j+1}^{j,j}+E_{j+1}^{j,j}\right)}^{-1}\left(D_{j+1}^{j+1,j}+E_{j+1}^{j+1,j}\right)J_{j+1|k}^{-1}\\ & \;\;\;\cdot \left(D_{j+1}^{j,j+1}+E_{j+1}^{j,j+1}\right){\left(J_{j|j}+D_{j+1}^{j,j}+E_{j+1}^{j,j}\right)}^{-1} \end{array}$$

Then using the matrix inversion lemma [24], the FIM $J_{j|k}$ is given by

(A15) $$\begin{array}{cc} J_{j|k} & =J_{j|j}+D_{j+1}^{j,j}+E_{j+1}^{j,j}-\left(D_{j+1}^{j+1,j}+E_{j+1}^{j+1,j}\right)[\left(D_{j+1}^{j,j+1}+E_{j+1}^{j,j+1}\right){\left(J_{j|j}+D_{j+1}^{j,j}+E_{j+1}^{j,j}\right)}^{-1}\\ & \;\;\;\cdot \left(D_{j+1}^{j+1,j}+E_{j+1}^{j+1,j}\right)+J_{j+1|k}{]}^{-1}\left(D_{j+1}^{j,j+1}+E_{j+1}^{j,j+1}\right) \end{array}$$

Substituting (4) into (A15), we have

(A16) $$\begin{array}{cc} J_{j|k} & =J_{j|j}+D_{j+1}^{j,j}+E_{j+1}^{j,j}-\left(D_{j+1}^{j+1,j}+E_{j+1}^{j+1,j}\right){\left(J_{j+1|k}+D_{j+1}^{j+1,j+1}+E_{j+1}^{j+1,j+1}-J_{j+1|j+1}\right)}^{-1}\\ & \;\;\;\cdot (D_{j+1}^{j,j+1}+E_{j+1}^{j,j+1}) \end{array}$$

This completes the proof.

## Appendix C. Proof of Theorem 3

From the assumptions that the noises are additive Gaussian white noises, we have

(A17) $$\begin{array}{cc} \; & \ln p\left(x_{k+1}|x_{k}\right)=c_{2}-\frac{1}{2}{\left(x_{k+1}-f_{k}\left(x_{k}\right)\right)}^{\prime }Q_{k}^{-1}\left(x_{k+1}-f_{k}\left(x_{k}\right)\right) \end{array}$$

where $c_{2}$ is a constant.

Thus, the partial derivatives of $\ln p(x_{k+1}|x_{k})$ are

(A18) $$\begin{array}{cc} \; & \;-\nabla_{x_{k}}\ln p\left(x_{k+1}|x_{k}\right)\\ \; & =\nabla_{x_{k}}[\frac{1}{2}\left({\left(x_{k+1}-f_{k}\left(x_{k}\right)\right)}^{\prime }Q_{k}^{-1}\left(x_{k+1}-f_{k}\left(x_{k}\right)\right)\right]\\ \; & =\nabla_{x_{k}}\frac{1}{2}\left[x_{k+1}^{\prime }Q_{k}^{-1}x_{k+1}-x_{k+1}^{\prime }Q_{k}^{-1}f_{k}\left(x_{k}\right)-f_{k}^{\prime }\left(x_{k}\right)Q_{k}^{-1}x_{k+1}+f_{k}^{\prime }\left(x_{k}\right)Q_{k}^{-1}f_{k}\left(x_{k}\right)\right)]\\ \; & =\nabla_{x_{k}}f_{k}^{\prime }\left(x_{k},\theta_{x}\right)Q_{k}^{-1}\left(f_{k}\left(x_{k}\right)-x_{k+1}\right) \end{array}$$

(A19) $$\begin{array}{cc} \; & \;-\Delta_{x_{k}}^{x_{k}}\ln p\left(x_{k+1}|x_{k}\right)\\ \; & =-\nabla_{x_{k}}\nabla_{x_{k}}^{\prime }\ln p\left(x_{k+1}|x_{k}\right)\\ \; & =\nabla_{x_{k}}\left[\left(f_{k}^{\prime }\left(x_{k}\right)-x_{k+1}^{\prime }\right)Q_{k}^{-1}\nabla_{x_{k}}f_{k}\left(x_{k}\right)\right]\\ \; & =\nabla_{x_{k}}f_{k}^{\prime }\left(x_{k}\right)Q_{k}^{-1}\nabla_{x_{k}}f_{k}\left(x_{k}\right)+\Delta_{x_{k}}^{x_{k}}f_{k}^{\prime }\left(x_{k},\theta_{x}\right)Q_{k}^{-1}f_{k}\left(x_{k}\right)-\Delta_{x_{k}}^{x_{k}}f_{k}^{\prime }\left(x_{k}\right)Q_{k}^{-1}x_{k+1}\\ \; & =\nabla_{x_{k}}f_{k}^{\prime }\left(x_{k}\right)Q_{k}^{-1}\nabla_{x_{k}}f_{k}\left(x_{k}\right)-\Delta_{x_{k}}^{x_{k}}f_{k}^{\prime }\left(x_{k},\theta_{x}\right)Q_{k}^{-1}\left(x_{k+1}-f_{k}\left(x_{k}\right)\right) \end{array}$$

Substituting (A19) into (3), we have

(A20) $$\begin{array}{cc} D_{k+1}^{k,k} & =E[-\Delta_{x_{k}}^{x_{k}}\ln p\left(x_{k+1}|x_{k}\right)]\\ \; & =E[\nabla_{x_{k}}f_{k}^{\prime }\left(x_{k}\right)Q_{k}^{-1}\nabla_{x_{k}}f_{k}\left(x_{k}\right)-\Delta_{x_{k}}^{x_{k}}f_{k}^{\prime }\left(x_{k}\right)Q_{k}^{-1}\left(x_{k+1}-f_{k}\left(x_{k}\right)\right)]\\ \; & =E[\nabla_{x_{k}}f_{k}^{\prime }\left(x_{k}\right)Q_{k}^{-1}\nabla_{x_{k}}f_{k}\left(x_{k}\right)] \end{array}$$

The remaining $D_{k+1}^{k+1,k}$, $D_{k+1}^{k+1,k+1}$, $E_{k+1}^{k,k}$, $E_{k+1}^{k+1,k}$ and $E_{k+1}^{k+1,k+1}$ can be obtained similarly. This completes the proof.

## Appendix D. Proof of Theorem 4

Applying the optimal filter [24] to the linear Gaussian systems (26)–(28), we have

(A21) $$\begin{array}{cc} & H_{k}^{*}=L_{k+1}F_{k}-\Psi_{k}L_{k} \end{array}$$

(A22) $$\begin{array}{cc} & R_{k}^{*}=L_{k+1}Q_{k}L_{k+1}^{\prime }+R_{k} \end{array}$$

(A23) $$\begin{array}{cc} & F_{k}^{*}=F_{k}-Q_{k}L_{k+1}^{\prime }{\left(R_{k}^{*}\right)}^{-1}H_{k}^{*} \end{array}$$

(A24) $$\begin{array}{cc} & Q_{k}^{*}=Q_{k}-Q_{k}L_{k+1}^{\prime }{\left(R_{k}^{*}\right)}^{-1}L_{k+1}Q_{k} \end{array}$$

(A25) $$\begin{array}{cc} & K_{k}=P_{k|k}{\left(H_{k}^{*}\right)}^{\prime }S_{k}^{-1} \end{array}$$

(A26) $$\begin{array}{cc} & S_{k}=H_{k}^{*}P_{k|k}{\left(H_{k}^{*}\right)}^{\prime }+R_{k}^{*} \end{array}$$

(A27) $$\begin{array}{cc} & P_{k|k+1}=P_{k|k}-K_{k}S_{k}K_{k}^{\prime }\\ \; & \;\;\;\;\;\;\;\;\;\;\overset{\left(A25\right)}{=}P_{k|k}-P_{k|k}{\left(H_{k}^{*}\right)}^{\prime }S_{k}^{-1}H_{k}^{*}P_{k|k} \end{array}$$

(A28) $$\begin{array}{cc} & P_{k+1|k+1}=F_{k}^{*}P_{k|k+1}{\left(F_{k}^{*}\right)}^{\prime }+Q_{k}^{*} \end{array}$$

For simplicity, we introduce

(A29) $$\left\{\begin{array}{cc} \; & B_{k}^{11}=D_{k+1}^{k,k}+E_{k+1}^{k,k}\\ \; & B_{k}^{12}=D_{k+1}^{k+1,k}+E_{k+1}^{k+1,k}={\left(B_{k}^{21}\right)}^{\prime }\\ \; & B_{k}^{22}=D_{k+1}^{k+1,k+1}+E_{k+1}^{k+1,k+1} \end{array}\right.$$

From (4) and the matrix inversion lemma [24], we have

(A30) $$\begin{array}{cc} J_{k+1|k+1}^{-1} & ={\left[B_{k}^{22}-B_{k}^{21}{\left(B_{k}^{11}+J_{k|k}\right)}^{-1}B_{k}^{12}\right]}^{-1}\\ \; & ={\left(B_{k}^{22}\right)}^{-1}+{\left(B_{k}^{22}\right)}^{-1}B_{k}^{21}{\left[B_{k}^{11}+J_{k|k}-B_{k}^{12}{\left(B_{k}^{22}\right)}^{-1}B_{k}^{21}\right]}^{-1}B_{k}^{12}{\left(B_{k}^{22}\right)}^{-1} \end{array}$$

Let

(A31) $$P_{k|k}=J_{k|k}^{-1}$$

The inverse of $B_{k}^{22}$ in (A30) can be rewritten as

(A32) $$\begin{array}{cc} {\left(B_{k}^{22}\right)}^{-1} & ={\left(Q_{k}^{-1}+L_{k+1}^{\prime }R_{k+1}^{-1}L_{k+1}\right)}^{-1}\\ \; & =Q_{k}-Q_{k}L_{k+1}^{{}^{\prime }}{\left(R_{k}^{*}\right)}^{-1}L_{k+1}Q_{k}\\ \; & \overset{\left(A24\right)}{=}Q_{k}^{*}. \end{array}$$

Rewrite ${\left(B_{k}^{22}\right)}^{-1}B_{k}^{21}$ in (A30) as

(A33) $$\begin{array}{cc} \; & \;{\left(B_{k}^{22}\right)}^{-1}B_{k}^{21}\\ \; & =\left(Q_{k}-Q_{k}L_{k+1}^{{}^{\prime }}{\left(R_{k}^{*}\right)}^{-1}L_{k+1}Q_{k}\right)\left(-Q_{k}^{-1}F_{k}-L_{k+1}^{{}^{\prime }}R_{k}^{-1}\Psi_{k}L_{k}\right)\\ \; & =-F_{k}-Q_{k}L_{k+1}^{\prime }R_{k}^{-1}\Psi_{k}L_{k}+Q_{k}L_{k+1}^{{}^{\prime }}{\left(R_{k}^{*}\right)}^{-1}L_{k+1}F_{k}\\ \; & \;+Q_{k}L_{k+1}^{{}^{\prime }}{\left(R_{k}^{*}\right)}^{-1}L_{k+1}Q_{k}L_{k+1}^{{}^{\prime }}R_{k}^{-1}\Psi_{k}L_{k}\\ \; & \overset{\left(A23\right)}{=}-F_{k}^{*}+Q_{k}L_{k+1}^{{}^{\prime }}{\left(R_{k}^{*}\right)}^{-1}\Psi_{k}L_{k}\\ \; & \;-Q_{k}L_{k+1}^{{}^{\prime }}{\left(R_{k}^{*}\right)}^{-1}\left[R_{k}^{*}-L_{k+1}Q_{k}L_{k+1}^{\prime }\right]R_{k}^{-1}\Psi_{k}L_{k}\\ & \overset{\left(A22\right)}{=}-F_{k}^{*}. \end{array}$$

Similarly, rewrite $B_{k}^{11}+J_{k|k}-B_{k}^{12}{\left(B_{k}^{22}\right)}^{-1}B_{k}^{21}$ as

(A34) $$\begin{array}{cc} \; & \;B_{k}^{11}+J_{k|k}-B_{k}^{12}{\left(B_{k}^{22}\right)}^{-1}B_{k}^{21}\\ \; & =J_{k|k}\;+\;F_{k}^{{}^{\prime }}Q_{k}^{-1}F_{k}\;+\;L_{k}^{{}^{\prime }}\Psi_{k}^{{}^{\prime }}R_{k}^{-1}\Psi_{k}L_{k}\;-\;\left(F_{k}^{\prime }Q_{k}^{-1}\;+\;L_{k}^{\prime }\Psi_{k}^{\prime }R_{k}^{-1}L_{k+1}\right)\\ \; & \;\cdot \left[Q_{k}-Q_{k}L_{k+1}^{{}^{\prime }}{\left(R_{k}^{*}\right)}^{-1}L_{k+1}Q_{k}\right]{\left(F_{k}^{\prime }Q_{k}^{-1}+L_{k}^{\prime }\Psi_{k}^{\prime }R_{k}^{-1}L_{k+1}\right)}^{\prime }\\ \; & =J_{k|k}\;+\;F_{k}^{{}^{\prime }}Q_{k}^{-1}F_{k}\;+\;L_{k}^{{}^{\prime }}\Psi_{k}^{{}^{\prime }}R_{k}^{-1}\Psi_{k}L_{k}\;-\;\left(F_{k}^{\prime }Q_{k}^{-1}\;+\;L_{k}^{\prime }\Psi_{k}^{\prime }R_{k}^{-1}L_{k+1}\right)\\ \; & \;\cdot Q_{k}{\left(F_{k}^{\prime }Q_{k}^{-1}+L_{k}^{\prime }\Psi_{k}^{\prime }R_{k}^{-1}L_{k+1}\right)}^{\prime }+\left(F_{k}^{\prime }Q_{k}^{-1}+L_{k}^{\prime }\Psi_{k}^{\prime }R_{k}^{-1}L_{k+1}\right)\\ \; & \;\cdot Q_{k}L_{k+1}^{{}^{\prime }}{\left(R_{k}^{*}\right)}^{-1}L_{k+1}Q_{k}{\left(F_{k}^{\prime }Q_{k}^{-1}+L_{k}^{\prime }\Psi_{k}^{\prime }R_{k}^{-1}L_{k+1}\right)}^{\prime }\\ \; & =J_{k|k}+F_{k}^{{}^{\prime }}Q_{k}^{-1}F_{k}+L_{k}^{{}^{\prime }}\Psi_{k}^{{}^{\prime }}R_{k}^{-1}\Psi_{k}L_{k}-F_{k}^{{}^{\prime }}Q_{k}^{-1}F_{k}-F_{k}^{\prime }L_{k+1}^{{}^{\prime }}R_{k}^{-1}\Psi_{k}L_{k}\\ \; & \;-L_{k}^{\prime }\Psi_{k}^{\prime }R_{k}^{-1}L_{k+1}Q_{k}L_{k+1}^{{}^{\prime }}R_{k}^{-1}\Psi_{k}L_{k}-L_{k}^{\prime }\Psi_{k}^{\prime }R_{k}^{-1}L_{k+1}F_{k}\\ \; & \;+\left[{\left(H_{k}^{*}\right)}^{\prime }L_{k}^{\prime }\Psi_{k}^{\prime }R_{k}^{-1}R_{k}^{*}\right){]{\left(R_{k}^{*}\right)}^{-1}\left[{\left(H_{k}^{*}\right)}^{\prime }L_{k}^{\prime }\Psi_{k}^{\prime }R_{k}^{-1}R_{k}^{*}\right)]}^{\prime }\\ \; & =J_{k|k}-{\left(H_{k}^{*}\right)}^{\prime }R_{k}^{-1}\Psi_{k}L_{k}-L_{k}^{\prime }\Psi_{k}^{\prime }R_{k}^{-1}L_{k+1}Q_{k}L_{k+1}^{{}^{\prime }}R_{k}^{-1}\Psi_{k}L_{k}-L_{k}^{\prime }\Psi_{k}^{\prime }R_{k}^{-1}L_{k+1}F_{k}\\ \; & \;+{\left(H_{k}^{*}\right)}^{\prime }{\left(L_{k+1}Q_{k}L_{k+1}^{{}^{\prime }}+R_{k}\right)}^{-1}H_{k}^{*}+L_{k}^{\prime }\Psi_{k}^{\prime }R_{k}^{-1}H_{k}^{*}\\ \; & \;+{\left(H_{k}^{*}\right)}^{\prime }R_{k}^{-1}\Psi_{k}L_{k}+L_{k}^{\prime }\Psi_{k}^{\prime }R_{k}^{-1}R_{k}^{*}R_{k}^{-1}\Psi_{k}L_{k}\\ \; & =J_{k|k}-L_{k}^{\prime }\Psi_{k}^{\prime }R_{k}^{-1}H_{k}^{*}+{\left(H_{k}^{*}\right)}^{\prime }{\left(R_{k}^{*}\right)}^{-1}H_{k}^{*}\\ \; & \;-L_{k}^{\prime }\Psi_{k}^{\prime }R_{k}^{-1}\left(L_{k+1}Q_{k}L_{k+1}^{{}^{\prime }}-R_{k}^{*}\right)R_{k}^{-1}\Psi_{k}L_{k}\\ \; & =J_{k|k}+{\left(H_{k}^{*}\right)}^{\prime }{\left(R_{k}^{*}\right)}^{-1}H_{k}^{*}. \end{array}$$

Thus, the inverse of $B_{k}^{11}+J_{k|k}-B_{k}^{12}{\left(B_{k}^{22}\right)}^{-1}B_{k}^{21}$ becomes

(A35) $$\begin{array}{cc} \; & \;{\left(B_{k}^{11}+J_{k|k}-B_{k}^{12}{\left(B_{k}^{22}\right)}^{-1}B_{k}^{21}\right)}^{-1}\\ \; & ={\left(P_{k|k}^{-1}+{\left(H_{k}^{*}\right)}^{\prime }{\left(R_{k}^{*}\right)}^{-1}H_{k}^{*}\right)}^{-1}\\ \; & =P_{k|k}-P_{k|k}{\left(H_{k}^{*}\right)}^{\prime }{\left[R_{k}^{*}+H_{k}^{*}P_{k|k}{\left(H_{k}^{*}\right)}^{\prime }\right]}^{-1}H_{k}^{*}P_{k|k}\\ \; & =P_{k|k}-P_{k|k}{\left(H_{k}^{*}\right)}^{\prime }S_{k}^{-1}H_{k}^{*}P_{k|k}\\ \; & \overset{\left(A27\right)}{=}P_{k|k+1} \end{array}$$

Then, from (A30), (A32), (A33) and (A35), the inverse of $J_{k+1|k+1}$ is

(A36) $$\begin{array}{cc} J_{k+1|k+1}^{-1} & =Q_{k}^{*}+F_{k}^{*}P_{k|k+1}{\left(F_{k}^{*}\right)}^{\prime }\overset{\left(A28\right)}{=}P_{k+1|k+1}. \end{array}$$

Using (6) and Corollary 1, the FIM for one-step prediction can be obtained as

(A37) $$\begin{array}{cc} J_{k+1|k} & =D_{k+1}^{k+1,k+1}-D_{k+1}^{k,k+1}{\left(D_{k+1}^{k,k}+J_{k|k}\right)}^{-1}D_{k+1}^{k+1,k}\\ \; & =Q_{k}^{-1}-Q_{k}^{-1}F_{k}{\left(F_{k}^{\prime }Q_{k}^{-1}F_{k}+J_{k|k}\right)}^{-1}F_{k}^{\prime }Q_{k}^{-1}\\ \; & ={\left(Q_{k}+F_{k}J_{k|k}^{-1}F_{k}^{\prime }\right)}^{-1} \end{array}$$

For the optimal one-step predictor, the MSE matrix $P_{k+1|k}$ is given by

(A38) $$\begin{array}{c} P_{k+1|k}=E\left[{\tilde{x}}_{k+1|k}{\tilde{x}}_{k+1|k}^{\prime }|y^{k}\right]=F_{k}P_{k|k}F_{k}^{\prime }+Q_{k} \end{array}$$

Then we have

(A39) $$\begin{array}{c} P_{k+1|k}\overset{\left(A31)(A37\right)}{=}J_{k+1|k}^{-1} \end{array}$$

Using (6) and Corollary 1, the FIM for two-step prediction can be written as

(A40) $$\begin{array}{cc} J_{k+2|k} & =D_{k+2}^{k+2,k+2}-D_{k+2}^{k+1,k+2}{\left(D_{k+2}^{k+1,k+1}+J_{k+1|k}\right)}^{-1}D_{k+2}^{k+2,k+1}\\ \; & =Q_{k+1}^{-1}-Q_{k+1}^{-1}F_{k+1}{\left(F_{k+1}^{\prime }Q_{k+1}^{-1}F_{k+1}+J_{k+1|k}\right)}^{-1}F_{k+1}^{\prime }Q_{k+1}^{-1}\\ \; & ={\left(Q_{k+1}+F_{k+1}J_{k+1|k}^{-1}F_{k+1}^{\prime }\right)}^{-1} \end{array}$$

For the optimal two-step predictor, one has

(A41) $$\begin{array}{cc} {\tilde{x}}_{k+2|k} & =x_{k+2}-{\hat{x}}_{k+2|k}\\ \; & =F_{k+1}\left(x_{k+1}-{\hat{x}}_{k+1|k}\right)+v_{k+1} \end{array}$$

and

(A42) $$\begin{array}{cc} P_{k+2|k} & =E[{\tilde{x}}_{k+2|k}{\tilde{x}}_{k+2|k}^{\prime }|y^{k}]\\ \; & =F_{k+1}P_{k+1|k}F_{k+1}^{\prime }+Q_{k+1} \end{array}$$

Then it follows from (A39), (A40) and (A42) that

(A43) $$\begin{array}{c} P_{k+2|k}=J_{k+2|k}^{-1} \end{array}$$

Similarly, we can prove that $P_{k+m|k}=J_{k+m|k}^{-1}$, m≥3. This completes the proof.

## Appendix E. Proof of Theorem 6

For the linear Gaussian systems (35) and (36), from [49], we have $P_{k|k}=J_{k|k}^{-1}$.

Using (6) and Corollary 2, the FIM for one-step prediction can be written as

(A44) $$\begin{array}{cc} J_{k+1|k} & =D_{k+1}^{k+1,k+1}-D_{k+1}^{k,k+1}{\left(D_{k+1}^{k,k}+J_{k|k}\right)}^{-1}D_{k+1}^{k+1,k}\\ \; & =Q_{k}^{-1}-Q_{k}^{-1}F_{k}{\left(F_{k}^{\prime }Q_{k}^{-1}F_{k}+J_{k|k}\right)}^{-1}F_{k}^{\prime }Q_{k}^{-1}\\ \; & ={\left(Q_{k}+F_{k}J_{k|k}^{-1}F_{k}^{\prime }\right)}^{-1} \end{array}$$

For the optimal one-step predictor, the MSE matrix $P_{k+1|k}$ is given by

(A45) $$\begin{array}{c} P_{k+1|k}=F_{k}P_{k|k}F_{k}^{\prime }+Q_{k} \end{array}$$

From (A44), (A45) and $P_{k|k}=J_{k|k}^{-1}$, we can obtain

(A46) $$\begin{array}{c} P_{k+1|k}=J_{k+1|k}^{-1} \end{array}$$

Using (6) and Corollary 2, the FIM for two-step prediction is given by

(A47) $$\begin{array}{cc} J_{k+2|k} & =D_{k+2}^{k+2,k+2}-D_{k+2}^{k+1,k+2}{\left(D_{k+2}^{k+1,k+1}+J_{k+1|k}\right)}^{-1}D_{k+2}^{k+2,k+1}\\ \; & =Q_{k+1}^{-1}-Q_{k+1}^{-1}F_{k+1}{\left(F_{k+1}^{\prime }Q_{k+1}^{-1}F_{k+1}+J_{k+1|k}\right)}^{-1}F_{k+1}^{\prime }Q_{k+1}^{-1}\\ \; & ={\left(Q_{k+1}+F_{k+1}J_{k+1|k}^{-1}F_{k+1}^{\prime }\right)}^{-1} \end{array}$$

For the optimal two-step predictor, one has

(A48) $$\begin{array}{cc} {\tilde{x}}_{k+2|k} & =x_{k+2}-{\hat{x}}_{k+2|k}\\ \; & =F_{k+1}\left(x_{k+1}-{\hat{x}}_{k+1|k}\right)+v_{k+1} \end{array}$$

and

(A49) $$\begin{array}{cc} P_{k+2|k} & =E[{\tilde{x}}_{k+2|k}{\tilde{x}}_{k+2|k}^{\prime }|Z^{k}]\\ \; & =F_{k+1}P_{k+1|k}F_{k+1}^{\prime }+Q_{k+1} \end{array}$$

Then it follows from (A46), (A47) and (A49) that

(A50) $$\begin{array}{c} P_{k+2|k}=J_{k+2|k}^{-1} \end{array}$$

Similarly, we can prove that $P_{k+m|k}=J_{k+m|k}^{-1}$, m≥3. This completes the proof.
